# Effect of chitosan-epoxy ratio in bio-based adhesive on physical and mechanical properties of medium density fiberboards from mixed hardwood fibers

**DOI:** 10.1038/s41598-024-55796-x

**Published:** 2024-03-01

**Authors:** Alireza Ashori, Anton Kuzmin

**Affiliations:** 1 Department of Chemical Technologies, Iranian Research Organization for Science and Technology (IROST), Tehran, Iran; 2 Department of Mechanization of Agricultural Products Processing, National Research Mordovian State University, 68 Bolshevistskaya Street, 430005 Saransk, Russia; 3 Scientific Laboratory of Advanced Composite Materials and Technologies, Plekhanov Russian University of Economics, 36 Stremyanny Ln, 117997 Moscow, Russia

**Keywords:** Chitosan, Epoxy, Mechanical properties, Dimensional stability, Gel time, Environmental chemistry, Environmental impact

## Abstract

Chitosan and bio-based epoxy resins have emerged as promising formaldehyde-free replacements for traditional urea–formaldehyde (UF) adhesives in engineered wood products. This study evaluated five chitosan-to-epoxy weight ratios (3:1, 2:1, 1:1, 1:2, 1:3) as adhesives for hot-pressing medium density fiberboards (MDF) using mixed hardwood fibers. Increasing the epoxy ratio reduced viscosity and gel time, facilitating spraying and fast curing. The density of the formulated MDFs increased with higher epoxy ratios, ranging from 679 kg/m^3^ for the 3:1 ratio to 701 kg/m^3^ for the 1:3 formulation, meeting the 500–900 kg/m^3^ density range specified in EN 323. The 1:3 epoxy-rich formulation enhanced modulus of rupture (MOR) to 31 MPa and modulus of elasticity (MOE) to 2392 MPa, exceeding the minimum requirements of 16 MPa and 1500 MPa set out in EN 310 and EN 316, respectively. Dimensional stability peaked at 5% thickness swelling for the 1:3 formulation after 24 h water soaking, fulfilling the < 25% requirement per EN 316. Internal bond strength reached a maximum of 0.98 MPa for the 3:1 chitosan-rich formulation, satisfying the 0.40 MPa minimum per EN 319. One-way ANOVA tests showed the adhesive ratio had a significant effect on mechanical properties and dimensional stability at 95–99% confidence levels. Duncan's multiple range test revealed the 1:3 ratio boards exhibited statistically significant improvements compared to untreated group. Overall, tailoring the ratios achieved well-balanced properties for MOR, MOE, and dimensional stability, demonstrating potential to replace UF resins.

## Introduction

A wood-based panel product, known as medium-density fiberboard (MDF), is created by combining lignocellulosic fibers with resin adhesives under heat and pressure. Traditionally, wood composites have utilized formaldehyde-based resins, such as urea–formaldehyde (UF), due to their strong bonding capabilities, fast curing process, and cost-effectiveness^1^. UF resin is widely used in the manufacturing of wood-based panels like MDF, particleboard, and plywood, accounting for 68% of global UF resin consumption in MDF and particleboard production and 23% in plywood production^2^. However, concerns have been raised regarding the release of formaldehyde from UF-bonded products, which can impact indoor air quality and human health. Formaldehyde is classified as a human carcinogen and can irritate the eyes, throat, and skin, as well as coughing and worsening of asthma symptoms^3–5^. As a result, there is a growing interest in developing formaldehyde-free, environmentally friendly wood adhesives. Some promising alternatives include:

- Bio-based polymers such as soy protein, chitosan, and lignin can form hydrogen bonds with wood fibers^6,7^.
- Starch adhesives derived from renewable agricultural sources^8^.
- Tannins extracted from natural materials, which exhibit phenolic self-condensation bonding^9^.
- Plant oil-derived epoxy resins that interact effectively with wood components^10^.
- Combinations of the above materials that optimize adhesive performance^11^.

In recent years, extensive research has been conducted to develop bio-based adhesives as alternatives to formaldehyde-emitting resins in engineered wood products. Renewable materials such as starch, lignin, tannin, cellulose nanofibril, soy protein, and chitosan have shown promise as wood adhesives^2,11^. Polylactic acid (PLA), polyhydroxyalkanoates (PHA), polyethylene furanoate (PEF), polybutylene succinate (PBS), and polytrimethylene terephthalate (PTT) are a group of synthetic biopolymers that are derived from natural materials through chemical synthesis processes^12^. Table 1 showcases a variety of sustainable raw materials that have the potential to be utilized in the formulation of adhesives^8^. However, their bonding strength, water resistance, and mechanical performance still need improvement to compete with conventional adhesives^4^. In addition, the availability of bio-based adhesive products for wood products is limited, and their high cost presents a challenge. While these adhesives can create premium-priced panels, they are not yet economically viable for mainstream panel production. To achieve a reasonable cost, the desired properties often require synthetic cross-linkers for existing and under-development adhesives^2^. Various strategies, including enzymatic modification, chemical grafting, and crosslinkers, have been employed to enhance the adhesion, moisture tolerance, and mechanical strength of bio-based adhesives^8,9,12^.

**Table 1:** Major renewable biopolymers, sources, and principal industrial uses. Reproduced with permission^8^.

Biopolymer	Source	Industrial uses
Cellulose	Plant-based materials (wood, cotton, hemp)	Paper products, textiles, packaging materials
Lignin	Plant-based materials (wood, agricultural residues)	Binders, adhesives, carbon fibers, filler material
Starch	Corn, potatoes, wheat, rice	Food packaging, biodegradable plastics, adhesives
Protein	Soy, wheat, corn, peas	Bioplastics, films, coatings, edible films
Oils and Waxes	Plants, seeds, fruits	Lubricants, coatings, waterproofing
Chitosan	Crustaceans shells (e.g., shrimp, crab)	Biomedical applications, wound dressings, drug delivery systems, and water treatment
Alginate	Brown seaweeds (e.g., kelp)	Food additives, pharmaceutical formulations, wound dressings, and biomedical applications
Pectin	Fruits, vegetables	Gelling agents, food additives, pharmaceuticals
Latex	Rubber trees, plants	Adhesives, sealants, gloves, foams
PLA	Corn starch, sugarcane, plant-based materials	Packaging materials, disposable cutlery, food containers, textiles
PHA	Microorganisms (fermentation of plant sugars or vegetable oils)	Packaging films, disposable items, agricultural mulch films, medical applications (drug delivery systems, tissue engineering)
PEF	Plant sugars (glucose, fructose)	Beverage bottles, films, fibers
PBS	Plant-based sugars (starch, glucose), succinic acid	Packaging materials, disposable cutlery, agricultural films, compostable bags
PTT	Plant-based materials (corn glucose, bio-based 1,3-propanediol)	Textiles, carpets, apparel

Renewable biopolymers like chitosan and epoxy resins have emerged as potential replacements for UF adhesives replacements^10^. Chitosan, derived from chitin, offers promising sustainable wood adhesion due to its hydroxyl and amine groups that readily bond with wood^6^. Widely used as adhesives and composites, epoxy resins exhibit excellent adhesion to wood through covalent bonding with cellulose^13^. Bio-based epoxies, derived from renewable plant oils, have gained interest as alternatives to standard petroleum-based epoxies that use bisphenol A and epichlorohydrin^14^. Epoxidized vegetable oils (EVOs) such as epoxidized soybean oil (ESBO) are synthesized from oils like soybean, canola, palm, and linseed through epoxidation with peracetic acid^15,16^. EVOs react with bio-based curing agents to form high-performance thermosets^17,18^, demonstrating excellent adhesion, strength, and resistance comparable to petroleum epoxies^19^.

**Figure 1:**
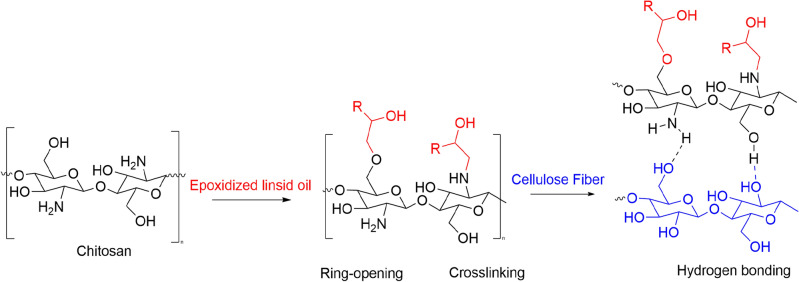
Schematic chemical reaction of chitosan, epoxy and wood fibers.

Chitosan contains abundant hydroxyl and amino functional groups along its molecular chain (Fig. 1). These polar groups can participate in specific intermolecular interactions that mediate adhesion to epoxy resins and lignocellulosic materials. Epoxy resins contain epoxide rings that can undergo ring-opening reactions with nucleophiles like amines and hydroxyls. This enables covalent crosslinking between epoxides and the functional groups on chitosan chains. The resulting chemical bonding provides intrinsic adhesion and load transfer between the two polymers. Cellulose fibers, the main component of wood and lignocellulosic substrates, also have abundant surface hydroxyl groups. These hydroxyls can hydrogen bond with the hydroxyl and amino groups on chitosan chains, allowing chitosan to coat and adhere to the hydrophilic fiber surfaces. Additionally, the amino groups on chitosan can react with carbonyl groups present in lignin via Schiff base formation, providing further interfacial bonding between chitosan adhesives and lignocellulosic materials. Combining these specific molecular level interactions—epoxide ring-opening, hydrogen bonding, and Schiff base formation—allows chitosan to act as an interface between the hydrophobic epoxy resin and the hydrophilic wood fibers. This promotes compatibility and adhesion between the resin matrix and lignocellulosic reinforcement, contributing to the overall performance of the composite system. Understanding these chemical bonding mechanisms provides molecular insights into the adhesion phenomena observed between chitosan, epoxy, and lignocellulosic substrates in this research^20^.

Combining chitosan with bio-epoxy resins offers a promising approach for the synergistic properties' enhancement, as observed in composites for coating and packaging applications^11,20,21^. Systematic optimization of chitosan-to-epoxy ratios, tailored explicitly for wood adhesives, could lead to UF resin replacements with well-balanced performance, manufacturing suitability, and environmental friendliness. However, despite the tremendous interest in bio-based adhesives, several inherent problems and challenges must be addressed for successful large-scale adoption and replacement of conventional adhesives. One major limitation is the lower bonding strength and inferior water resistance of many biopolymers compared to traditional formaldehyde-based resins^18,22,23^. Additionally, their higher viscosity and susceptibility to thermal degradation during hot pressing can restrict processability^2^. To overcome these challenges, it is essential to minimize properties' variability by implementing purification and modification processes for biopolymers sourced from biological materials^6^. Further optimization of curing reactions and achieving a balanced hydrophobic/hydrophilic characteristic is necessary to ensure adequate moisture resistance^10^. Understanding the complex interfacial interactions between renewable adhesives and lignocellulosic substrates is crucial^11^. Additionally, developing cost-effective and eco-friendly modification strategies is essential for industrial viability. While there have been recent advances in the field, fully harnessing the potential of biopolymers as adhesives will require continued research and development efforts to overcome these remaining challenges.

The current study aims to develop an optimized chitosan-epoxy adhesive formulation and fabrication process as a viable alternative to formaldehyde-emitting resins for medium-density fiberboard (MDF) production. The weight ratio of chitosan-to-epoxy was selected as the primary variable parameter, with five levels (3:1, 2:1, 1:1, 1:2, and 1:3). These ratios were systematically evaluated to assess their impact on vital adhesive properties such as gel time and viscosity. MDF boards were then fabricated using the different adhesive formulations, and a comprehensive analysis was performed to evaluate important physico-mechanical properties, including modulus of rupture (MOR), modulus of elasticity (MOE), internal bond strength (IBS), thickness swelling (TS), and water absorption (WA). The collected data was carefully analyzed to determine the optimal chitosan-to-epoxy ratio that exhibits suitable adhesive characteristics and enhances the properties of the boards compared to conventional UF-based resins. The findings from this study contribute to the development of an optimized chitosan-epoxy formulation and fabrication process for producing environmentally friendly, UF-free MDF boards.

## Materials and methods

### Materials

Chitosan with an 85% degree of acetylation and an average molecular weight of 32 kDa was obtained from Sigma-Aldrich (USA) (CAS No. 9012-76-4). Analytical-grade acetic acid (CAS No. 64-19-7), sodium hydroxide (CAS No. 1310-73-2), ethanol (CAS No. 64-17-5), ammonium chloride (CAS No. 12125-02-9), and glycerol (plasticizer, CAS No. 56-81-5) were obtained from Merck KGaA, Darmstadt, Germany to ensure purity standards suitable for laboratory research. Merck sources chemicals from reputable producers that meet the highest grades for scientific applications. Certificates of analysis were provided with the purchased products guaranteeing a minimum purity of 99.5%. Epoxidized linseed oil (ELO), the bio-based epoxy resin, was acquired from Arkema Co. (France) (CAS No. 8016-11-3). The VESTAMIN® IPD brand diethyl toluene diamine (DETDA) hardener solution (CAS No. 68479-98-1) was obtained from Evonik Industries, Germany. All chemicals were utilized as received without undergoing any additional purification steps.

A commercial-grade UF resin was supplied by Khazar Caspian Wood Industry Co. (Amol, Iran). This UF resin had a density of 1.3 g/cm^3^, a solid content of 60 wt%, viscosity of 420 cP, and a gel time of 70 s. The resin had a formaldehyde to urea (F/U) mole ratio of 1.4 and was catalyzed with 1% ammonium chloride. The CAS number of this resin is 9011-05-6.

The wood fibers utilized in this investigation were obtained from Khazar Caspian Wood Industry Co. (Amol, Iran) as a blend of hardwood species. The hardwood species included maple (*Acer spp.*), birch (*Betula spp.*), aspen (*Populus spp.*), beech (*Fagus spp.*), oak (*Quercus spp.*), and ash (*Fraxinus spp.*). These fibers were generated through a thermo-mechanical pulping process at the mill. Due to the mixed nature of the furnish, it was not feasible to determine the precise proportions of each hardwood species.

### Preparation of chitosan-epoxy adhesives

The following procedure was used to synthesize the chitosan-epoxy bio-resins. First, 5 g of chitosan powder was added to 100 mL of 1% v/v acetic acid solution at a concentration of 5% w/v. The solution was stirred at 300 rpm for 24 h using a mechanical stirrer to form a homogeneous gel. Next, epoxidized linseed oil (ELO), a renewable epoxy source, was slowly added to the chitosan gel under vigorous stirring. The weight ratios of chitosan to ELO used were C3:E1, C2:E1, C1:E1, C1:E2, and C1:E3. The stirring continued for 1 h to mix the components. To enhance the flexibility of the resulting resins, glycerol was added at a content of 5% based on the weight of chitosan. The mixture was stirred for 30 min to ensure uniform distribution of the plasticizer. In order to initiate the crosslinking reaction between the amine groups of chitosan and the epoxy groups, the pH of the mixture was modified to a range of 8–9 using sodium hydroxide. For the rapid production of chitosan-epoxy-based MDF panels, DETDA was employed as a hardener. DETDA, an aromatic amine, can form stable crosslinked structures with the epoxy rings. Approximately 1.5% DETDA by weight of the resin was utilized to expedite the gelation and curing process.

### MDF fabrication

The MDF panels used in the experiment were manufactured to precise dimensions of 400 mm × 400 mm × 16 mm. These panels had an average density of 0.76 g/cm^3^. The fabrication process of the MDF boards involved the following steps. First, the hardwood fibers were mixed to create a uniform blend. These fibers were then subjected to oven-drying at 105 °C until they reached a 4–6% moisture content. Next, the chitosan-epoxy adhesive was manually sprayed onto the dried fibers. The adhesive was applied at a resin content of 10% based on the weight of the oven-dried fibers. It is important to mention that the adhesive was diluted to a lower viscosity to allow it to flow through a spray nozzle. Alcohol as solvent was used to make the gel more liquid for spraying. In addition, the adhesive was applied with an airbrush tool that relies on compressed air to atomize and evenly spray on the surface of wood fibers. The airbrush pressures range from 20 to 30 psi. The chitosan-epoxy adhesive-coated fibers were manually filled into a forming box and subjected to pre-pressing to create a mat. The pre-pressing was accomplished by applying a pressure of 1.0 MPa. Subsequently, the mat of resin-coated fibers was transferred to a laboratory hot press machine, set to a temperature of 160 °C and a pressure of 4 MPa. The hot press process lasted for 7 min. To control the thickness of the panels and prevent excessive compression, stoppers or thickness spacers were employed during the hot-pressing procedure. For comparison purposes, another fiber mat was prepared as the control using UF resin. The UF resin was also applied at a resin content of 10% based on the weight of the oven-dried fibers. The hot-pressing conditions for the UF-based boards were the same as those mentioned earlier. Triplicate panels were manufactured for each adhesive formulation to ensure consistency and reliability. After fabrication, the panels were conditioned at a temperature of 20 °C and a relative humidity of 65% for two weeks before undergoing testing.

### Testing

#### Determination of viscosity and gel time

The viscosity of the synthesized chitosan-epoxy adhesive was determined following the ASTM D2196-10:2010 standard test method using a rotational viscometer^21^. This test method outlines the procedure for measuring the rheological properties of non-Newtonian materials using a cone-plate viscometer geometry. The adhesive sample was loaded between the stationary plate and the rotating cone spindle of the viscometer fixture. The gap between the cone tip and plate was set at 0.121 cm, as specified for resin samples in the method. The test temperature was maintained at 25 °C ± 0.1 °C using the attached Peltier system. Shear rate ramps were programmed from 0.1/s to 1000/s with 5 points per decade and a dwell time of 10 s per data point. Three test repetitions were performed to account for variability.

The gel time of the chitosan-epoxy resin was determined using the test tube method outlined in ASTM D2471-99:2019^22^. This method monitors the transition from liquid to solid during the curing process. Approximately 3 g of resin was filled into glass test tubes (13 mm diameter, 100 mm length) and immersed in a silicone oil bath preheated to 140 ± 1 °C. At regular intervals, the tubes were removed and tilted 90° to check for the flow of the resin inside the tilted tube. The time taken from insertion into the oil bath until the resin gelled and stopped moving upon tilting was noted as the gel time. Cessation of flow on tilting indicated the resin had reached its gel point. Three repetitions were performed and the average gel time was reported.

#### Density and moisture content

The density and moisture content of the MDF boards were evaluated following the guidelines in EN 322:1993^23^ and EN 323:1993^24^ standards. Rectangular specimens were cut from the boards, weighed, and thickness measured. The samples were conditioned at 20 ± 2 °C and a relative humidity of 65 ± 5% until constant mass was achieved. The final mass was measured and density calculated by dividing mass by volume. Moisture content (MC) was determined as the percentage of mass loss after conditioning. Three replicates were tested for each formulation to ensure accuracy.

#### Thickness swelling and water absorption

The MDF boards' thickness swelling and water absorption were evaluated following the EN 317:1993 standard^25^. Rectangular specimens measuring 50 mm × 50 mm were immersed in water at 20 °C for specified intervals^30^. Post-immersion, the thickness was measured to assess swelling. Water absorption was calculated as the percentage increase compared to the initial oven-dry weight. Three replicates were conducted for each formulation to ensure accuracy and account for natural variation. To ensure accuracy and reliability, three replicates were tested for each formulation.

#### Mechanical properties

The MDF boards' modulus of rupture (MOR), modulus of elasticity (MOE), and internal bond strength (IBS) were determined following the guidelines in EN 310:1993^26^ and EN 319:1993^27^ standards. Prior to testing, the specimens were conditioned at 20 ± 2 °C and 65 ± 5% RH until constant mass was achieved. The testing was conducted using an Instron universal testing machine (Norwood, MA, USA) with a 5 kN load cell. Specimens of 250 mm × 50 mm were prepared for the three-point bending test. The samples were placed on the testing machine with a span of 200 mm. A crosshead speed of 5 mm/min was employed during the test. The testing software recorded the load–deflection data, which enabled the calculation of both MOR and MOE. The same universal testing machine was used, this time in the tensile mode. Specimens with 50 mm × 50 mm were utilized, and a crosshead speed of 2 mm/min was employed.

#### Field emission scanning electron microscopy

The interface morphology of the samples was visualized using a TESCAN MIRA3 field emission scanning electron microscope (TESCAN Orsay Holding, Brno, Czech Republic). Images were acquired at a magnification of 500x. Before analysis, a thin gold coating was applied to the samples to enhance conductivity and imaging quality. The FE-SEM was operated at an accelerated voltage of 15 kV to achieve optimal imaging conditions. This voltage setting ensures sufficient electron beam energy for sample penetration and generation of high-resolution images.

#### Statistical analysis

The statistical analysis was carried out utilizing version 22 of the SPSS software program. The data underwent a one-way analysis of variance (ANOVA) to identify any significant differences within the groups, with confidence intervals set at the 95% and 99% levels. To determine the significant differences between and among the groups, Duncan's multiple range test (DMRT) was employed.

## Results and discussion

### Viscosity and gel time

Using the data presented in Table 2, it can be observed that the viscosity values exhibited a decreasing trend with increasing epoxy content in the adhesive formulations. For instance, the viscosity dropped from 1270 cP for the 3:1 chitosan-to-epoxy ratio (board type C3:E1) to 525 cP for the 1:3 ratio (board type C1:E3). This decrease in viscosity can be attributed to the difference in molecular weights between chitosan (200–300 kDa) and the epoxy resin oligomers (~ 700 Da). At higher chitosan ratios, the adjoint polymers occupy more volume, leading to increased resistance to flow and higher viscosity. However, as the epoxy content increases, the polymer chain length and molecular weight decrease. This decrease in chain length results in lower entanglement and inter-chain interactions, consequently leading to lower viscosity. It is worth noting that for spraying applications, the optimal viscosity range for wood adhesives is typically less than 1000 cP^28^. The lower end of this range is suitable for blending methods. The decreasing viscosity trend with higher epoxy content aligns with the findings reported by Hashim et al.^29^. They attributed the viscosity reduction to the lower molecular weight and chain entanglement of epoxy. Similarly, Kamarian and Song^30^ reported a 40% lower viscosity for a 1:2 ratio adhesive compared to a 2:1 ratio, although their specific values differed due to variations in molecular weights.

**Table 2:** Effect of chitosan-to-epoxy ratio on viscosity and gel time.

Resin type	Chitosan:Epoxy ratio	Viscosity (cP)	Gel time (s)
C3:E1	3:1	1,270 ± 104	176 ± 9
C2:E1	2:1	1,146 ± 98	157 ± 11
C1:E1	1:1	860 ± 111	126 ± 17
C1:E2	1:2	643 ± 68	92 ± 15
C1:E3	1:3	525 ± 47	84 ± 13
UF	–	318 ± 58	19 ± 21

As the epoxy ratio increased, the gel times of the adhesive formulations decreased. For example, the C3:E1 formulation gelled in 176 s, while the C1:E3 formulation gelled even lower in just 84 s. This phenomenon can be attributed to the epoxy component's high reactivity and crosslink density^31^. Epoxy exhibits rapid polycondensation through the ring-opening reaction of epichlorohydrin groups, accelerating the gelation process. With higher epoxy ratios, epoxy functional groups are more available, promoting faster crosslinking and reducing the gel time. However, it is important to note that excessively high epoxy content can lead to premature gelation, limiting the working time for adhesive application. The reduction in gel time at higher epoxy ratios is consistent with findings in the literature. The accelerated gelation associated with higher epoxy content is advantageous for fast-curing applications. However, it is essential to consider the limitations of excessively fast gelling. Zolghadr et al.^32^ recommended limiting the epoxy content to avoid overly rapid gelation.

### Density

Density is an important property of MDF as it affects their overall weight and strength. The density of MDF boards varied depending on the chitosan-to-epoxy ratios from 679 to 701 kg/m^3^. Lower-density boards are generally desirable as they are lighter and offer improved workability and ease of handling. However, it's important to note that the control sample (UF-MDF) had a density of 688 kg/m^3^, suggesting that the chitosan-epoxy bonded boards had comparable or only slightly higher densities. Figure 1a shows that as the epoxy ratio was increased, the density of the adhesive formulations exhibited a linear increase, ranging from 679 kg/m^3^ for the C3:E1 to 701 kg/m^3^ for the C1:E3 board type. This trend agrees with the findings reported by Ferdosian et al.^10^, who observed an increase in density with higher epoxy content. The gradual enhancement in density closely corresponds to the typical range of 600–800 kg/m^3^ specified for standard MDF products according to EN 622-5:2010^33^. This observed trend can be due to the higher specific gravity of fully cured epoxy (1.1–1.4 g/cm^3^) compared to the lower density of the chitosan biopolymer (1.03 g/cm^3^) as reported by Talaei et al.^34^. At higher epoxy ratios, the composite density is primarily influenced by the higher intrinsic density of epoxy. The control MDF bonded with UF resin had a density of 671 kg/m^3^. It is worth noting that all the chitosan-epoxy density values met the EN 323:1993 standard for MDF (500–900 kg/m^3^)^24^. The increasing density range of 679–701 kg/m^3^ with higher epoxy ratios provides new quantitative data on achievable densities for these specific chitosan-to-epoxy formulations, aligning with previous studies that reported an increase in density with higher epoxy content. Increasing density with higher epoxy ratios helps enhance mechanical properties, such as MOR and MOE. This is achieved through better stress transfer between the wood fibers and the polymer matrix, facilitated by superior fiber-matrix interaction enabled by the higher-density composite structure.

Additionally, the higher density restricts swelling and water absorption by reducing available space for moisture ingress. The intrinsic hydrophobicity of epoxy resin also contributes to reducing water uptake. The density increase partially contributes to reduce moisture content by limiting moisture diffusion into the composite structure. Furthermore, the non-polar nature of the epoxy resin resists moisture absorption. The high reactivity and crosslink density of epoxies accelerates the curing process, leading to reduced gel times. Therefore, the trends of increasing density, viscosity, and gel time with higher epoxy content can be explained by the density, rheological, and curing characteristics of the epoxy resin system.

#### Moisture content

Moisture content is a critical property that affects the dimensional stability and durability of wood-based composites. In this study, the moisture content of the MDF panels bonded with chitosan-epoxy adhesives varied with different chitosan-to-epoxy ratios. The values ranged from 6.4 to 8% for the various ratios tested. With an increasing epoxy ratio in the adhesive formulations, the moisture content of the composites progressively decreased. The sample with the highest chitosan content (3:1 mixing ratio) exhibited the maximum moisture absorption at 8 ± 0.8%, which was 1.6% higher than the control sample bonded with UF resin. As the chitosan content decreased from 3:1 to 1:3, the moisture content also reduced. Even the MDF sample bonded with the lowest chitosan content (1:3) had a moisture content of 4.8 ± 0.3%, which was 1.3% lower than the control.

The moisture absorption in the composites was directly proportional to the chitosan content, indicating chitosan's role in increasing moisture absorption compared to neat epoxy. This trend can be attributed to epoxy's hydrophobic nature and chitosan's hydrophilicity. Epoxy, with its non-polar groups, resists moisture absorption, while chitosan, with its hydroxyl groups, attracts water molecules^35^. At higher epoxy levels, moisture uptake is restricted. It is interesting to note that despite the similar chemical structure of chitosan and cellulose, chitosan absorbs more water. This is due to chitosan's more open and porous structure, additional hydrogen bonding through amino groups, higher solubility, swelling capacity, and lower crystallinity than cellulose. All moisture content values remained within the maximum limit of 10% specified by the EN 323:1993 standard^24^. The reduction in moisture content with higher epoxy content has significant implications for the mechanical and physical properties of the composites. Lower moisture content improves mechanical properties such as modulus of rupture and elasticity by reducing plasticization and swelling effects that can deteriorate the fiber-matrix interface.

#### Thickness swelling and water absorption

**Figure 2:**
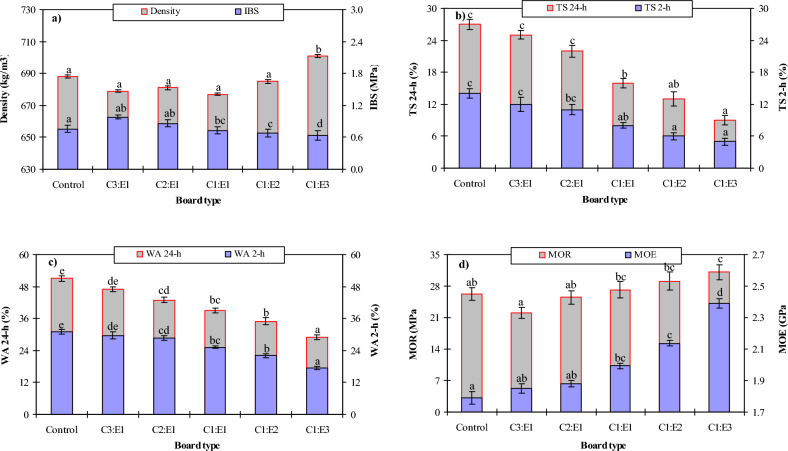
Physical and mechanical characteristics of the MDFs: (**a**) Density and internal bond strength (IBS), (**b**) Thickness swelling (TS), (**c**) Water absorption (WA), (**d**) Modulus of rupture (MOR) and modulus of elasticity (MOE).

Thickness swelling measures the dimensional stability of the MDF boards when exposed to moisture. It indicates the extent to which the boards expand or swell in thickness due to water absorption. The thickness swelling values ranged from 5 to 12% for different ratios after 2 h of exposure to moisture (Fig. 2b). Lower values of thickness swelling indicate better resistance to moisture absorption and improved dimensional stability, which is important for applications where the boards may be exposed to humidity or wet environments. As the epoxy ratio increased, there was a significant decrease in thickness swelling and water absorption in the adhesive formulations. The thickness swelling decreased from 25% (1:3) to 5% (3:1), while water absorption dropped from 47 to 17.4%, respectively. In comparison, the control sample bonded with UF resin exhibited a thickness swelling of 14% (2 h) and 26.7% (24 h), as well as water absorption of 31.1% (2 h) and 51% (24 h). Tan and Chow^29^ reported similar reductions in thickness swelling and water absorption. They concluded that the hydrophilicity and moisture sensitivity of chitosan contribute to higher swelling and absorption compared to the hydrophobic epoxy polymer.

The observed lower swelling and absorption with higher epoxy contents align with the maximum limits specified by the EN 317:1993 standard^25^. This allows for a maximum thickness swelling of 25% and water absorption of 45%^30^. Water absorption is a measure of the ability of the MDF panels to absorb water. It represents their susceptibility to moisture uptake, affecting their dimensional stability and durability. In this study, the water absorption values ranged from 17.4 to 29.7% for different ratios after 2 h of water exposure (Fig. 2c). Compared to the UF control, the chitosan-epoxy boards exhibited lower thickness swelling and water absorption, particularly at lower epoxy ratios. The reduced thickness swelling and water absorption with increased epoxy content contribute to improved mechanical performance by limiting degradation of the fiber-matrix interface through swelling and plasticization effects. The decreased swelling and absorption correlate with higher viscosity and slower curing times. The hydrophilic nature of chitosan leads to greater moisture uptake, lower viscosity, and faster gelation than the hydrophobic epoxy^17–19^. Additionally, the duration of the test impacts the extent of moisture uptake. The swelling and absorption values at 2 h were lower compared to 24 h for all board types, indicating an increasing water ingress over time, especially for compositions with higher chitosan content. The lower thickness swelling and water absorption positively affect the mechanical properties of the adhesive formulations. These reductions also relate to the rheological and curing behaviors of the formulations.

### Mechanical properties

#### MOR

The MOR is a measure of the strength of the MDF boards. It represents the maximum stress that the boards can withstand before fracturing or breaking under a bending load. In this study, the modulus of rupture varied with different chitosan-to-epoxy ratios. The values ranged from 22.07 to 31.13 MPa for different ratios (Fig. 1d). Higher values of MOR indicate greater strength and load-bearing capacity, which are desirable for various applications where structural integrity is crucial. The control samples bonded with UF resin exhibited a typical MOR of 26.3 MPa, MOE of 1791 MPa, and IBS of 0.76 MPa, consistent with standard industrial UF-bonded MDF^36^. In contrast, the chitosan-epoxy MDF boards showed progressive enhancements in these mechanical properties with increasing epoxy resin content, although the exact results depended on the specific formulation. The MOR increased from 22.07 to 31.13 MPa with higher epoxy ratios, indicating improved strength. However, lower epoxy formulations showed inferior MOR compared to UF-MDF, highlighting the need for sufficient epoxy to reinforce the wood-chitosan matrix^12,36^.

#### MOE

The MOE is a measure of the stiffness or rigidity of the MDF boards. It indicates their ability to resist deformation under an applied load. The MOE values ranged from 1791 to 2392 MPa for different ratios. Higher values of MOE suggest that the boards are stiffer and less prone to bending or flexing, which can be advantageous in applications that require dimensional stability and resistance to bending or sagging. The MOE increased from 1851 to 2392 MPa with higher epoxy content, surpassing the MOE at high epoxy levels but showing lower values for chitosan-dominant formulations (Fig. 2d). The reductions in MOR and MOE at lower epoxy levels can be attributed to the lower stiffness, strength, and adhesion of chitosan compared to epoxy. Chitosan has lower mechanical stiffness and strength due to extensive hydrogen bonding between chains, while epoxy forms highly crosslinked rigid networks. Higher proportions of chitosan dilute the reinforcing effect of stiffer epoxy chains, resulting in decreased MOR and MOE. Additionally, chitosan has weaker adhesion to wood due to hydrogen bonding than epoxy's covalent bonding with cellulose^36^.

#### IBS

The IBS is a measure of the adhesion and bonding quality between the individual wood fibers within the MDF boards. It represents the ability of the boards to resist separation or delamination. In this study, the internal bond strength varied from 0.64 to 0.98 MPa for different ratios (Fig. 2a). In contrast to MOR and MOE, the IBS showed an increasing trend with higher chitosan content in the composites. The sample with the highest chitosan content exhibited a maximum IBS of 0.98 MPa. In comparison, the control sample had an IBS of 0.64 MPa, which was lower than most of the tested samples, except for one specific formulation. The internal bond strength depends more on the interfacial adhesion between the chitosan and epoxy phases. As the chitosan content decreases, there are fewer chitosan-epoxy interfacial areas for bonding. Chitosan likely forms hydrogen bonds and mechanical interlocks with epoxy due to its hydroxyl and amino groups. With less chitosan, there are fewer interactions and, hence, lower bonding. On the other hand, MOR and MOE depend more on the overall composite structure and macroscale reinforcement. The higher modulus of epoxy compensates for the lower chitosan content at higher ratios. The intrinsic stiffness and brittleness of epoxy may also contribute to improved MOR and MOE but worse interfacial bonding as its proportion increases. Chitosan's flexibility allows stress dissipation between phases, enhancing interfacial bond strength. The intrinsic stiffness and brittleness of epoxy may also contribute to improved MOR and MOE but worse interfacial bonding as its proportion increases. Chitosan's flexibility allows stress dissipation between phases, enhancing interfacial bond strength. With less chitosan, this dissipation is reduced^37,38^.

The combination of chitosan and epoxy in the composite system leads to improved mechanical properties compared to UF-bonded panels. The chemical reactions and hydrogen bonding interactions between chitosan, epoxy, and wood fibers are crucial in enhancing the composite's interfacial adhesion and overall performance. The specific mechanical properties, such as MOR, MOE, and IBS, are influenced by the ratio of chitosan to epoxy and the resulting balance between stiffness, strength, and interfacial bonding.

### Statistical analysis

One-way ANOVA revealed the adhesive ratio significantly influenced the mechanical properties of the MDF boards (Table 3). The 1:3 epoxy formulation yielded boards with markedly higher modulus of rupture (31.13 MPa) than the 3:1 ratio (22.07 MPa) at 95% confidence per Duncan's test (Fig. 2d). This formulation also produced boards with superior stiffness, evidenced by the maximum modulus of elasticity (2392 MPa) compared to all other ratios at 99% confidence (Fig. 2d). However, the 3:1 ratio gave the highest internal bond strength (0.98 MPa), significantly exceeding the 1:2 and 1:3 ratios at 99% confidence (Fig. 2a).

**Table 3:** One-way ANOVA results for effect of adhesive ratio on mechanical and density properties.

Source	MOR		MOE		IBS		Density	
	*F*	*P*	*F*	*P*	*F*	*P*	*F*	*P*
A	4.67	0.013*	16.42	0.000**	16.42	0.000**	4.36	0.017*

*A* adhesive ratio, *F* F value, *ns* not significant, *Significant difference at the 5% level (*p* ≤ 0.05%), **Significant difference at the 1% level (*p* ≤ 0.01%).

The adhesive ratio likewise had a highly significant effect (*p* < 0.001) on dimensional stability properties (Table 4). Boards made with the 1:3 formulation showed the least 2-h thickness swelling (5%) compared to the 3:1 and 2:1 ratios at 99% confidence (Fig. 2b). This formulation maintained the minimum 24-h thickness swelling (12%) versus all ratios at *p* < 0.001. Additionally, the 24-h thickness swelling values remained below the maximum limit of 25% specified in EN 316 for wood-based panels exposed to moisture cycling^39^. The 1:3 ratio boards absorbed the lowest moisture (17.4%) after 2 h, significantly lower than other formulations at *p* < 0.001 (Fig. 2c). They retained the minimum water uptake (29.7%) after 24 h based on Duncan's test. In summary, the 1:3 epoxy-rich adhesive optimized modulus of rupture, modulus of elasticity, and dimensional stability. However, maximum internal bond strength resulted from chitosan-dominant 3:1 formulation. Statistical analyses quantitatively validated the significant effect of tailoring chitosan-epoxy ratios on enhancing key MDF board properties.

**Table 4:** One-way ANOVA results for effect of adhesive ratio on dimensional stability.

Source	TS (2-h)		TS (24-h)		WA (2-h)		WA (24-h)	
	*F*	*P*	*F*	*P*	*F*	*P*	*F*	*P*
A	8.47	0.001**	22.84	0.000**	59.34	0.000**	21.55	0.000**

*A* adhesive ratio, *F* F value, *ns* not significant, *Significant difference at the 5% level (*p* ≤ 0.05%), **Significant difference at the 1% level (*p* ≤ 0.01%).

### Morphological studies

**Figure 3:**
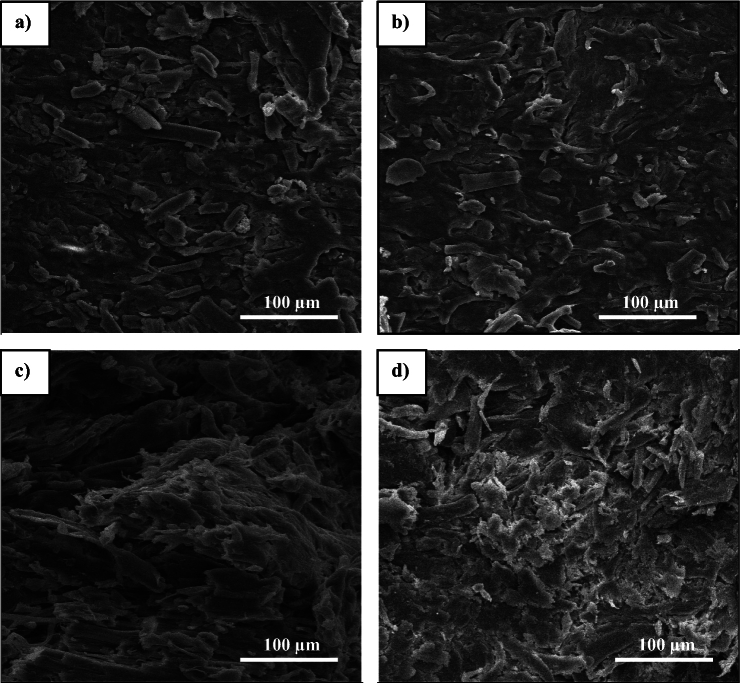
Tensile fracture surfaces of the samples: (**a**) C3:E1, (**b**) C2:E1, (**c**) C1:E2, and (**d**) C1:E3.

The main objective of this research was to establish a strong interface between wood fibers and chitosan-epoxy resins in MDF boards. Figure 3 illustrates the tensile fracture surfaces of different ratios of chitosan:epoxy, providing valuable insights. In the case of samples C3:E1 and C2:E1 (Fig. 3a and b), the SEM analysis revealed uneven fracture surfaces with many broken fibers and microvoids. These characteristics indicate weak interfacial bonding between the wood veneer and resin matrix. Conversely, notable disparities were observed in the fiber-resin film interfaces in samples C1:E2 and C1:E3 (Fig. 3c and d). A uniform resin film was observed covering the fibers, and there were only a few instances of fiber pullouts and microvoids. This suggests improved compatibility between the fibers and resin, resulting in a less distinct interfacial boundary. Overall, these findings demonstrate that incorporating C1:E3 enhances the interfacial bonding and compatibility between wood fibers and the resin matrix, as evidenced by SEM observations. This strengthened interface contributes to the overall mechanical properties and performance of the MDF boards.

## Conclusions

This study analyzed the effect of varying chitosan-to-epoxy weight ratios on the performance of formaldehyde-free MDFs. A ratio of 1:3 chitosan to epoxy was found to optimize key properties. At this formulation, the MDF boards demonstrated a density of 701 kg/m^3^, modulus of rupture of 31.13 MPa, modulus of elasticity of 2392 MPa, and internal bond strength of 0.84 MPa. These results met or exceeded the benchmarks set by conventional UF-bonded MDF boards. Additionally, the 1:3 ratio formulation showed thickness swelling of 5% and water absorption of 17.4% after 2 h exposure. These results were superior to the control UF-MDF which exhibited 14–26.7% thickness swelling and 31.1–51% water absorption, indicating enhanced moisture resistance. One-way ANOVA tests showed the adhesive ratio had a significant effect on mechanical properties and dimensional stability at 95–99% confidence levels. Duncan's multiple range test revealed the 1:3 ratio boards exhibited statistically significant improvements in modulus of rupture, modulus of elasticity, thickness swelling and water absorption compared to other formulations. Overall, tailoring the chitosan-to-epoxy ratio to a minimum of 1:3 ratio balanced performance metrics across density, mechanical properties, moisture uptake, and dimensional stability. This enables complete replacement of UF resins with renewable chitosan-epoxy adhesives in MDF fabrication. Further testing and optimization are still required to build on these promising findings before large-scale manufacturing implementation. However, this work demonstrates the potential for high performance, formaldehyde-free, eco-friendly MDF boards using bio-based adhesives.

## Data Availability

The datasets used and/or analyzed during the current study available from the corresponding author on reasonable request.
